# Supplementary material for: Triggering Chinese lecturers’ intrinsic work motivation by value-based leadership and growth mindset: Generation difference by using multigroup analysis
Source: PLoS One. 2024 Mar 27;19(3):e0297791. doi: 10.1371/journal.pone.0297791 (PMC10971668; doi:10.1371/journal.pone.0297791)
Supplement: S1 File — (DOCX) [file pone.0297791.s001.docx]

The adapted items to measure intrinsic work motivation, value-based leadership, and growth mindset:

|  | Questions |
| --- | --- |
|  | ***Intrinsic work motivation*** |
| IWM1 | You make efforts getting involved in your job is because you have fun doing your job. |
| IWM2 | You make efforts getting involved in your job is because what you do in your work is exciting. |
| IWM3 | You make efforts getting involved in your job is because the work you do is interesting. |
|  | ***Value-based leadership*** |
| VBL1 | Your leader is truthful, honest, and displays moral behavior. |
| VBL2 | Your leader has healthy self-confidence and self-esteem. |
| VBL3 | Your leader does not lose sight of his or her goals or compromise on his or her principles. |
| VBL4 | Your leader has an inspiring vision. |
| VBL5 | Your leader finds ways to communicate his or her vision to his or her followers. |
| VBL6 | Your leader inspires trust and hope in his or her followers. |
| VBL7 | Your leader has the loyalty of the followers. |
| VBL8 | Your leader has a willingness to serve. |
| VBL9 | Your leader listens to his or her followers. |
| VBL10 | Your leader encourages dissenting opinion among his or her closest advisers. |
| VBL11 | Your leader is committed to the moral principle of respect for the followers. |
| VBL12 | Your leader includes the people affected in the change process. |
| VBL13 | Your leader is clear about his or her own beliefs e.g. assumptions about human nature, the role of the organization, the measurement of performance, etc. |
| VBL14 | Your leader listens to the needs, ideas, and aspirations of his or her followers and responds to them within the context of his or her well-developed systems of belief in the appropriate fashion. |
| VBL15 | Your leader has ideas. |
| VBL16 | Your leader shares information with his or her followers. |
| VBL17 | Your leader fosters a sense of community. |
| VBL18 | Your leader creates a consistent system of rewards, structure, process, and communication. |
| VBL19 | Your leader is committed to a principle of opportunity, giving all followers the chance to make a contribution to the organization. |
|  | ***Growth mindset*** |
| GW1 | No matter who you are, you can change your intelligence a lot. |
| GW2 | You can always greatly change how intelligent you are. |
| GW3 | No matter how much intelligence you have, you can always change it quite a bit. |
