# Supplementary material for: Triggering Chinese lecturers’ intrinsic work motivation by value-based leadership and growth mindset: Generation difference by using multigroup analysis
Source: PLoS One. 2024 Mar 27;19(3):e0297791. doi: 10.1371/journal.pone.0297791 (PMC10971668; doi:10.1371/journal.pone.0297791)
Supplement: Attachment — Submitted filename: comments on intrinsic motivation.docx [file pone.0297791.s002.docx]

This study attempts to address the effects of value-based leadership and growth mindset on the intrinsic work motivation of Chinese lecturers as well as investigate the generational differences between the effects of Millennials and their predecessors. The manuscript is overall well written, presents a clear theoretical framework, methodology, findings and discussions for the study. However, it left some to be desired and needs minor revision, especially on its methodology section. See comments below:

Since the study is about lecturers’ motivation, the term lecturer should be defined and explained. I notice that in a later section, “faculty member” is also used. Are they similar or different? Are these lecturers non tenure-track or tenure-track? Full-time or part-time? What are their primary responsibilities in the universities? Also, are these lecturers from different universities you surveyed have the same responsibilities? Because these statuses would affect their motivation, I think it needs to be addressed clearly.

In the literature review section, I would like to see some new studies on the motivation, as the context could change over time. Looking at the first paragraph on Page 3, some studies were done in 1972, 2000, 2001. Though I do think the Confucianism does influence how people think and behave, individuals may have different interpretations of the traditional thoughts. I would like to see if there are any regional, generational, and gender differences on desiring “ high moral character and a fairly low requirement for wealth”.

On page 3, influencing factors of intrinsic work motivation are summarized and categorized, but no citations have been provided to different theories. Please add.

For the section on “ influencing factors of ….” , I would recommend reorganize it to focus on value-based leadership rather than “organizational variables” . The authors are trying to make a connection between organizational values and values of leaders, but such connection is not explicitly stated and explained.

For the methodology section, the sample size of 518 lecturers are categorized as younger (40 years old) and older (>40 years old), but in the literature review, the millennials are defined as people who were born between 1979-1990, so I would think the old-young category should be based on the definition. Is that correct? Why do the authors select 40 as the dividing line?

I am not clear about what type of universities the authors selected to survey their lecturers. Are all of them from the same university? In China, a 985 university is quite different from a college in a small city? Without knowing this type of information, it would be difficulty to draw a valid conclusion.

As far as the measure are concerned, I found quite interesting that the authors used MWMS and Dweck’s three-item scale to do the study. The authors kept mentioning that the Chinese context is quite different from the western context to rationalize the importance and significance of the study, why didn’t they develop their own measures to reflect the Chinese context, but rather use the measures developed by western scholars? Please provide the rationales. If the measures are updated version, please provide any information on what have been updated and why?
