# Supplementary material for: Triggering Chinese lecturers’ intrinsic work motivation by value-based leadership and growth mindset: Generation difference by using multigroup analysis
Source: PLoS One. 2024 Mar 27;19(3):e0297791. doi: 10.1371/journal.pone.0297791 (PMC10971668; doi:10.1371/journal.pone.0297791)
Supplement: Attachment — Submitted filename: Response to Reviewers.docx [file pone.0297791.s003.docx]

**Reviewer #3:**

| **No** | **General Comments** | **Reply to reviewer** |
| --- | --- | --- |
| 1 | Introduction section: while the authors present a well-structured introduction, I suggest expanding the discussion of relevant contributions (at the bottom of page 2), and complementing it with the summary of the key findings, while articulating how these findings expand existing knowledge in a meaningful way. | Thank you for your comments.  We have revised the introduction to include a more comprehensive discussion of the relevant contributions and clearly articulate how our findings expand existing knowledge in a meaningful way. This will help readers better understand the significance of our research within the field. |
| 2 | Underpinning Theory section: While the authors list several theories that are relevant to the subject matter of this paper, the key claims of each of these theories need to be explained in somewhat greater detail. Specifically, the “first” and “second” categories of relevant theoretical developments (on page 3) require additional explanations. This should allow the authors to better position their study relative to existing conceptual stipulations. It will also allow the authors to better explain the novelty and theoretical contribution of this study. | Thank you for your comments.  We have revised the Underpinning Theory section to provide a more comprehensive overview of these theories and their key claims. |
| 3 | Literature Review section: While the authors present a good and relevant literature review, this discussion is missing important recent studies that should be acknowledged. Specifically, generational differences have been recently examined as moderators on various cognitive and behavioral associations in organizational and institutional contexts (Mahmoud, Berman, Reisel, Fuxman, & Hack-Polay, 2023). Furthermore, since the study examines the relevance of cultural norms and values in China, I suggest citing relevant recent literature on cognition, the development of cultural norms and attributes, and the consequent behavioral outcomes (Berman, Mudambi, & Shoham, 2022). | Thank you for your suggestions.  We have revised the literature review by adding the recent studies you mentioned. |
| 4 | Results section: While I consider the utilized methodology to be appropriate and robust, I suggest providing “economic” interpretation of several key coefficients that are discussed in this section. In particular, the economic meaning of the differences in the path coefficients between two groups found in the Multigroup Analysis (MGA) should be explained, in order to better articulate the relevance of the generational differences as a moderator. | We appreciate the suggestion. We have added a discussion on the MGA result with respect to path coefficients between the two groups. |
| 5 | Implications and Limitations section: Additionally, while the authors consider generational differences as a moderating variable in the study, other potentially relevant demographic variables are not explicitly considered. For example, gender has been recently found to be an important moderating variable between cognitive and behavioral outcomes (Mahmoud, Berman, Grigoriou, & Solakis, 2023). Similarly, individuals’ “self-efficacy” has been recently examined as a determinant of certain managerial behaviors in higher education (Mahmoud, Berman, Reisel, Fuxman, Grigoriou, & Tehseen, 2023). I suggest acknowledging these limitations in the related section. | We appreciate your suggestions to acknowledge these limitations in the related section. To address this limitation, we have included a discussion, acknowledging the omission of these relevant variables and their potential impact on the findings. |

References:

Mahmoud, A. B., Berman, A., Reisel, W., Fuxman, L., & Hack‐Polay, D. (2023). Examining generational differences as a moderator of extreme‐context perception and its impact on work alienation organizational outcomes: Implications for the workplace and remote work transformation. Scandinavian Journal of Psychology.

Berman, A., Mudambi, R., & Shoham, A. (2022). Linguistic structures and innovation: A behavioral approach. Journal of International Management, 28(4), 100943.

Mahmoud, A. B., Berman, A., Grigoriou, N., & Solakis, K. (2023). Investigating gender as a moderator of extreme-context perception effects on behavioural tendencies towards fashion brands on Instagram in West Africa. Journal of Marketing Communications, 1-23.

Mahmoud, A. B., Berman, A., Reisel, W., Fuxman, L., Grigoriou, N., & Tehseen, S. Entrepreneurial Marketing Intentions and Behaviours among Students: Investigating the Roles of Entrepreneurial Skills, Entrepreneurial Self-efficacy, and Family Business Exposure.
